# FTH1 protects against osteoarthritis by MAPK pathway inhibition of extracellular matrix degradation

**DOI:** 10.1186/s12891-024-07411-3

**Published:** 2024-04-12

**Authors:** Zhikun Yuan, Lingfeng Yang, Yanhui Li, Xuming Li, Changgui Peng, Jianying Pan, Daozhang Cai

**Affiliations:** 1 Department of Orthopedics, Orthopedic Hospital of Guangdong Province, Academy of Orthopedics·Guangdong Province, The Third School of Clinical Medicine, The Third Affiliated Hospital, Southern Medical University, Guangzhou, China; 2 Department of Orthopedics, Shijie Hospital of Dongguan City, Dongguan, China; 3 Department of Pathology, Shijie Hospital of Dongguan City, Dongguan, China

**Keywords:** Osteoarthritis, ECM, Cartilage, Ferroptosis

## Abstract

**Objective:**

Ferritin heavy chain 1 (FTH1) is an important subunit of ferro-storing proteins and is indispensable for iron metabolism. Though it has been extensively studied in numerous organs and diseases, the relationship between FTH1 and osteoarthritis (OA) is unclear.

**Design:**

Primary murine chondrocytes and cartilage explants were treated with FTH1 siRNA for 72 h. Mice were injected with adenovirus expressing FTH1 after destabilized medial meniscus (DMM) surgery. These approaches were used to determine the effect of FTH1 expression on the pathophysiology of OA.

**Results:**

FTH1 expression was down regulated in OA patients and mice after DMM surgery. Knock down of FTH1 induced articular cartilage damage and extracellular matrix degradation in cartilage explants. Further, over expression of FTH1 reduced the susceptibility of chondrocytes to ferroptosis and reversed decrements in SOX9 and aggrecan after DMM surgery. Moreover, FTH1 relieved OA by inhibition of the chondrocyte MAPK pathway.

**Conclusion:**

This study found FTH1 to play an essential role in extracellular matrix degradation, ferroptosis, and chondrocytes senescence during OA progression. Further, injection of adenovirus expressing FTH1 may be a potential strategy for OA prevention and therapy.

**Supplementary Information:**

The online version contains supplementary material available at 10.1186/s12891-024-07411-3.

## Introduction

Osteoarthritis (OA) is a common degenerative disease of individuals over 50 years of age. OA usually leads to degeneration of the knee joint and progressive loss of motion as the condition progresses [1]. Based on conservative estimates, OA affects up to 240 million individuals worldwide [2], placing serious financial burden on individuals and governments [3]. Numerous research investigations have been carried out to clarify the specific mechanistic basis for OA pathogenesis. Chondrocytes are mature articular cartilage cells and their dysfunction is crucial to the pathogenesis of OA, with restoration of normal chondrocyte function essential to OA treatment [4]. Empty cartilage lacunae are a histological characteristic of OA, indicating that the physiological status of chondrocytes is intimately connected to the OA pathological process [5, 6]. Iron is the most abundant trace element in the human body, and is necessary for maintenance of normal growth and development [7]. However, excessive free cellular iron participates in the Fenton reaction, forming reactive oxygen species (ROS) such as hydroxyl radicals. Accumulated ROS peroxidizes membrane lipids, by a process known as ferroptosis, which causes loss of cell function and cell death [8]. Ferroptosis is a novel form of iron-dependent cell death due to iron accumulation within cells and as such is different from other known forms of regulated cell death [7]. Increased lipid hydroperoxide levels and iron overload are defining characteristics of ferroptosis, whch induce caspase-independent and necrosome-independent cell death [9]. SLC7A11 and GPX4, as important defense factors of ferroptosis, have been demonstrated in an increasing number of studies for their ability to delay the development of ferroptosis, and as important markers for assessing the susceptibility of cells to ferroptosis [10, 11].

Ferritin heavy chain 1 (FTH1), a subunit of ferritin, is a spherical heteropolymer that can store excess cellular iron to maintain intracellular iron homeostasis [12]. FTH1 exhibits ferroxidase activity, which catalyzes transformation of cytoplasmic Fe2 + into the ferric form (Fe3 +) [13]. Ferritin FTH1 reduces the formation of lipid peroxides by storage of excess free intracellular iron, which decreases ROS production, reduces impaired cellular function, and mitigates ferroptosis [14]. Ferroptosis occurs in chondrocytes during OA, resulting in extracellular matrix (ECM) degradation and chondrocytes senescence [15]. Each of which contribute to OA development. It is important to note that the relationship between FTH1 and the development of OA is unknown.

FTH1 is down regulated in the chondrocytes of OA patients. We hypothesized that FTH1 is involved in the development of OA. To support this hypothesis, we demonstrated that FTH1 deficiency decreased the expression of anabolic markers and lead to cartilage loss in primary murine chondrocytes and 3-week-old mouse explants. Further, over expression of FTH1 reduced chondrocyte sensitivity to ferroptosis and protected articular cartilage from destabilized medial meniscus surgery (DMM). Furthermore, FTH1 was shown to contribute to OA by modulation of the MAPK signaling pathway. These results suggest that FTH1 may be an alternative and effective therapeutic target for OA treatment.

## Materials and methods

### Human samples

The Third Affiliated Hospital Ethics Committee of Southern Medical University approved the study. Human samples were collected after obtaining informed consent.

Five tibial plateaus from OA patients who underwent total knee replacement surgery were collected. Damaged joint surfaces of tibial plateau are classified as the OA group. In contrast, the smooth surface is ranked as the negative control group. Furthermore, all these specimens were stored at -80℃ for later experiment.

### Mice

We purchased 8-week-old male C57BL/6J mice from Beijing Vital River Laboratory Animal Technology (Beijing, China), and C57 male mice were raised to 12-week-old before right knee joint destabilized medial meniscus surgery (DMM). All mice were cared for under the institution’s policies on animal use and care. To medically establish a OA mouse model, firstly we intraperitoneal injection of 60 mg/kg pentobarbital to anesthesia mice, and then cut open the skin and joint cavity of the mice and cut off the ligament between the medial meniscus and tibial plateau. The sham surgery group had a procedure that opened and exposed the right knee's tissues after intraperitoneal injection of 60 mg/kg pentobarbital. After DMM surgery and sham surgery, 4 µL PBS (vehicle)/mouse 4µL Ad-FTH1/mouse (Han heng Biology, Wuhan, China) was administered by intra-articular injection twice per week. The right knee joint were collected 4 weeks after DMM and sham surgery (*n* = 5 per group). Then samples were fixed with 4%paraformaldehyde for 24 h, decalcified for a month, dehydrated, embedded and sectioned.

### Cartilage explants

We euthanize Three-week-old male C57 mice and collected tibial plateaus cartilage explants from them. Micro tweezers were used to cut off tibial plateaus from proximal tibia. Cartilage explants were grown in 12-well plates for 3 days in DMEM/F12 with 10% fetal bovine serum and 1% penicillin and streptomycin after treated with FTH1 si-RNA.

### Cells

Primary murine chondrocytes were derived from tibial plateaus and ribcage of 6-day-old C57BL/6J mice and were cultured in DMEM/F12 containing 10% fetal bovine serum added with 1% Penicillin–Streptomycin in the 6cm petri dishes for later study. An in vitro OA chondrocyte model was produced by treating primary murine chondrocytes with 5 ng/mL interleukin-1 (IL-1) (R&D systems) for 48 h.

### siRNA transfection

Primary murine chondrocytes were seeded in 6-well plates before siRNA transfection, about 20 × 10^4^ cells per well. After 48 h, FTH1 si-RNA(100 nM) and FTH1 si-control(100 nM) were transfected into chondrocytes with lipofectamine 3000 (2μL/mL) (Thermo Fisher Scientific) for 24 h, and change the medium on the following day. FTH1 si-RNA(100 nM) and FTH1 si-control(100 nM) were transfected into cartilage explants with lipofectamine 3000 (2μL/mL) (Thermo Fisher Scientific) for 72 h.

### The qRT-PCR

TRIzol reagent was used to extract total RNA from primary mouse chondrocytes and was reverse transcription into complementary cDNA with the aid of a Reverse Transcription Master Mix. (Vazyme Biotech, Nanjing, China) LightCycler® 96 Instrument—Roche was used to performing qPCR assay. Here is a list of mouse primer sequences:

FTH1:Forward5’-GCCGAGAAACTGATGAAGCTGC-3’,Reverse5’-GCACACTCCATTGCATTCAGCC-3’;GPX4:Forward5’-CCTCTGCTGCAAGAGCCTCCC-3’,Reverse5’CTTATCCAGGCAGACCATGTGC-3’;GAPDH:Forward5’-AGGTCGGTGTGAACGGATTTG-3’,Reverse5’-TGTAGACCATGTAGTTGAGGTCA-3’;SOX9:Forward5’-GAGCCGGATCTGAAGAGGGA-3’,Reverse5’-GCTTGACGTGTGGCTTGTTC-3’; Aggrecan:Forward5’-TCCACATCAGAAGAGCCATAC-3’,Reverse5’-AGTCAAGGTCGCCAGAGG-3’;col2a1:Forward5’-CTTAGGACAGAGAGAGAAGG-3’,Reverse5’-ACTCTGGGTGGCAGAGTTTC-3’;P16:Forward5’-ACATCAAGACATCGTGCGATATT-3’,Reverse5’-CCAGCGGTACACAAAGACCA-3’;P21:Forward5’-CCTGGTGATGTCCGACCTG-3’, Reverse5’-CCATGAGCGCATCGCAATC -3’;

### Reactive oxygen species assay

After the chondrocytes were treated with FTH1 siRNA, they were removed from the medium and added to the DCFH-DA (Beyotime, S0033S) working solution and incubated for 30 min at 37 °C, protected from light. And then the cells were washed three times with serum-free medium. The images were captured and analyzed by fluorescence microscopy (OLYMPUS BX51).

### Western blot analysis

Cells cultured in 6-well dishes were, after lysed with 200μL of radioimmunoprecipitation assay (RIPA) buffer, and then added protease inhibitor and phosphatase inhibitor 2μL. Heating at 100 degrees for 15 min after additionally add 40μL 5 × Loding buffer(Vazyme Biotech, Nanjing, China). And then the protein samples additionally addgel electrophoresis (SDS-PAGE), and then were transferred to polyvinylidene difluoride (PVDF) membranes. Membranes were incubated for 1 h at room temperature in shaking bed with 5% skim milk solution. And then membranes incubated for 16 h at 4℃ in shaking bed with primary antibodies. And then washed the membranes three times with TBST solution, 5 min per time. Finally the membranes were incubated for 1 h at room temperature in shaking bed with 5% skim milk solution and secondary antibodies diluted in it. Protein bands were exposed by FDbio-Dura ECL(FDbio science, Hangzhou, China). Antibodies used for western blotting were: Ferritin Heavy Chain Rabbit mAb( ABclonal, 1:1000, A19544), rabbit anti-P21 (Abcam, 1:2000, ab109520), SOX9 Rabbit mAb (ABclonal, 1:1000, A19710), ERK1/2 Polyclonal antibody (Proteintech 1:1000, 11257–1-AP), Phospho-ERK1/2 Polyclonal antibody (Proteintech 1:1000, 28733–1-AP).

### Histology staining

Slides were deparaffinized and rehydrated after heated at 65℃ for 2 h. And then slides were washed with PBS solution for three times, 5 min per time. And then we used Safranin O-fast green dye solution and hematoxylin and eosin dye solution for staining.

### Immunohistochemical (IHC) staining

Slides were deparaffinized and rehydrated after heated at 65℃ for 2 h. And then Put slides in the box filled with TE9.0, heated them with microwave for 2 min and cool naturally to room temperature. After slides were washed with PBS solution for three times, 5 min per time, covered with hydrogen peroxide solution for 10 min, and then washed them three times again. After that, slides were incubated with primary antibodies for 16 h at 4 °C.After cooling naturally to room temperature, slides were washed with PBS solution for three times, 5 min per time. And then slides were incubated with second antibodies for 1 h at room temperature. After slides were washed with PBS solution for three times, 5 min per time, the slides for IHC were stained with hematoxylin and diaminobenzidine. Finally, took pictures and analyzed them.

### Immunocytochemistry (ICC)

Primary murine chondrocytes were seeded on cover slips in a 12-well plate, and were treated with si-FTH1(100 nM,TSINGKE), 5 ng/ml IL-1β and Ad-FTH1(10^9^PFU/ml, HANBIO, Wuhan, China) for 48 h. After washed 3 times with PBS, cells were fixed with 4% paraformaldehyde for 15 min. After three washed with PBS for 3 min each, cells were treated with 0.2% Triton X-100 (MACKLIN, Shanghai, China) for 20 min. The cells were then covered with 10% normal bovine serum (Solarbio, Beijing, China) for 1 h at room temperature. The cells were then treated with primary antibodies for 16 h at 4 °C. The fluorescent secondary antibody for ICC were applied for 1 h at room temperature. Then the cells were stained with DAPI (Invitrogen, USA). Finally, images were took by fluorescence microscope(OLYMPUS BX51). Adobe Photoshop 2021 was used to to eliminate fluorescence bleed-through and analyse the images. Antibodies used for ICC were: Ferritin Heavy Chain Rabbit mAb (ABclonal, 1:100, A19544), Phospho-ERK1/2 Rabbit Polyclonal antibody (Proteintech 1:200, 28733–1-AP), Goat anti-Rabbit IgG Secondary Antibody, Alexa Fluor™594 (Invitrogen, 1:500, A-11012).

### Statistical analyses

Data are displayed as mean SD. Unpaired Student's t-tests were used in experiments comparing two groups of data. *P*-values < 0.05 were regarded as significant. GraphPad Prism 8 was used to analyze all of the data.

## Results

### FTH1 is down-regulated in chondrocytes of OA patients and OA mice

**Fig. 1:**
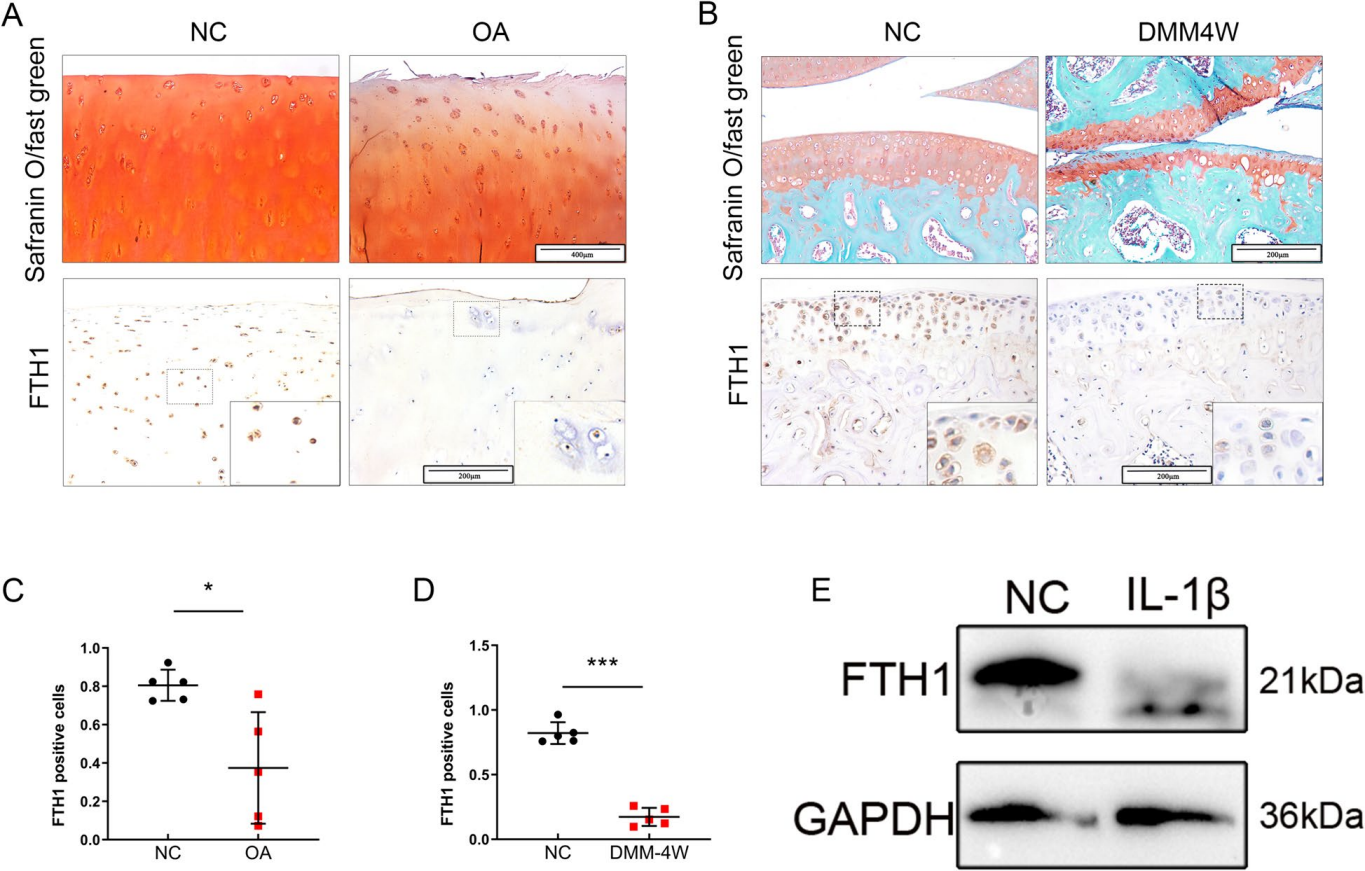
Loss of chondrocytes expressing FTH1 in OA patients and DMM mice. **A** Safranin O and Fast Green staining (upper row) and immunostaining of FTH1 (lower row) of OA cartilage. Scale bar: 400 µm(upper row) and 200 µm(lower row);** B** Safranin O and Fast Green staining (upper row) and immunostaining of FTH1 (lower row) of DMM mice cartilage. Scale bar: 200 µm; **C** Percentage of positive cells of FTH1 in human OA cartilage. *n* = 5 per group; **D** Percentage of positive cells of FTH1 in DMM mice cartilage *n* = 5 per group; **E** Immunoblotting of FTH1 in primary murine chondrocytes which treated with 5 ng/ml IL-1βfor 48 h

Initially, the expression of FTH1 in the cartilage of OA patients and the relationship of FTH 1 expression to OA were determined. Immunohistochemistry (IHC) demonstrated FTH1 levels to be lower in severely damaged OA patient cartilage (derived from total knee replacement tissue) compared to smooth and only slightly damaged cartilage (Fig. 1A, B). A DMM-induced OA animal model was used to assess FTH1 expression throughout OA development. FTH1 was significantly down regulated in DMM-induced OA mice with severe cartilage destruction compared to normal cartilage of control mice (Fig. 1 C, D). Western blot analysis demonstrated FTH1 to be significantly down regulated in in vitro primary murine chondrocytes treated with IL-1β for 48 h. (Fig. 1 E) Collectively, FTH1 expression was found to be decreased in OA cartilage, suggesting that FTH1 plays an essential role in the etiology of OA.

### FTH1 deficiency results in a chondrocyte senescence phenotype

**Fig. 2:**
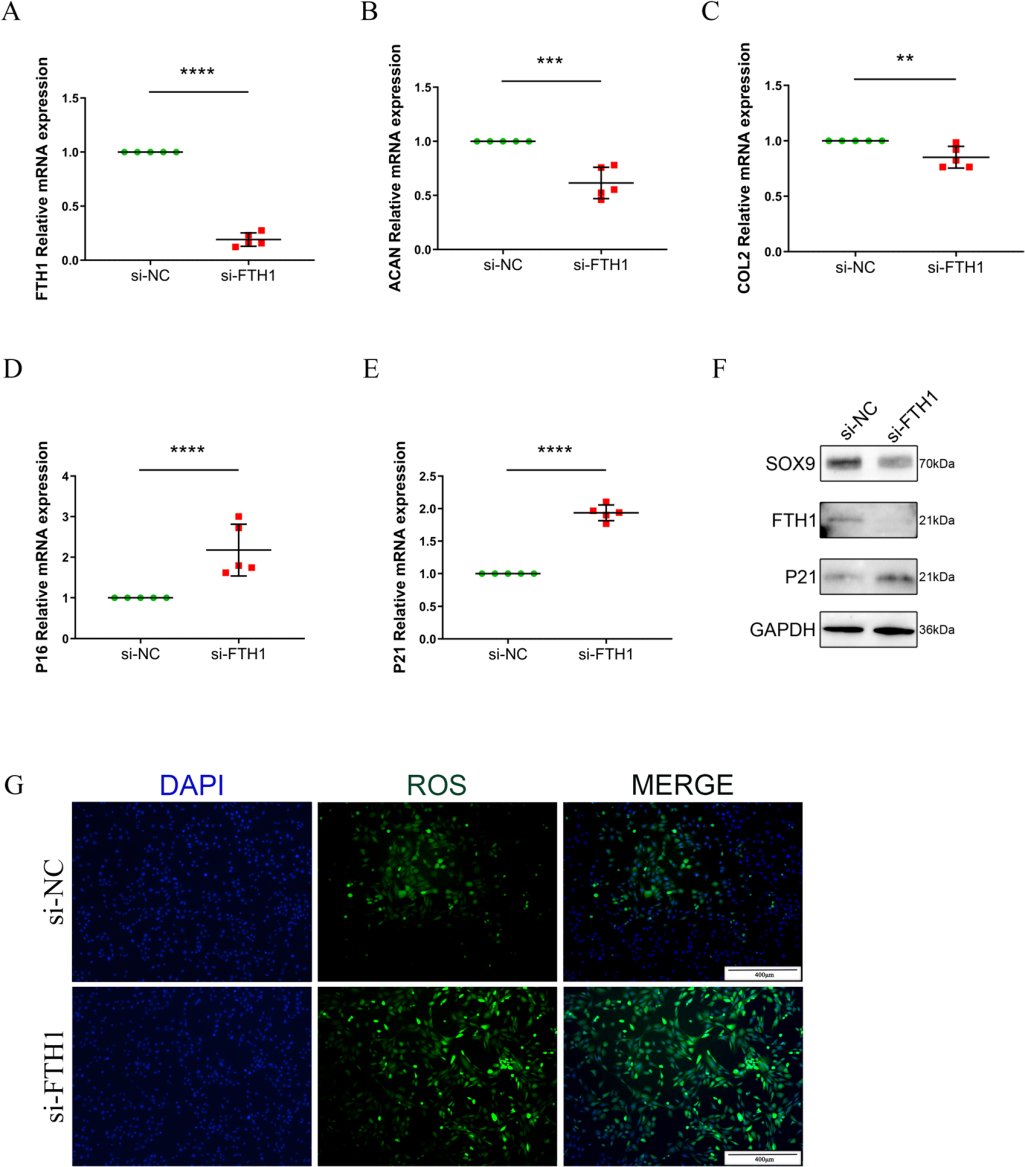
FTH1 deficiency leads to chondrocytes senescence and ECM degradation**. A** Relative mRNA expression level of FTH1 in primary murine chondrocytes which treated with FTH1 siRNA for 48 h; Relative mRNA expression level of aggrecan (**B**), collagen type II (**C**), P16 (**D**) and P21 (**E**) in primary murine chondrocytes which treated with FTH1 siRNA for 48 h; *n* = 5,*P < 0.05, **P < 0.01, ***P < 0.001,****P < 0.0001; (**F)** Immunoblotting of FTH1, SOX9 and P21 in primary murine chondrocytes which treated with FTH1 siRNA for 48 h; (G) ROS level of chondrocytes, after transfection with FTH1 siRNA or si-NC. Scale bar: 400 µm

To evaluate the contribution of FTH1 to OA, FTH1 si-RNA was used to knock down FTH1 expression in primary murine chondrocytes. FTH1 expression was significantly decreased after transfection of chondrocytes with FTH1 siRNA (Fig. 2 A). Quantitative reverse transcription PCR (qRT-PCR) of chondrocytes after knock down of FTH1 demonstrated down regulation of cartilage synthesis markers, aggrecan (ACAN) and collagen type II (Fig. 2 B, C). Indicators of chondrocyte senescence, P16 and P21, were markedly elevated (Fig. 2 D, E). Western blot analysis demonstrated that defective FTH1 expression resulted in a chondrocyte senescence phenotype, which is known to exacerbate ECM degradation and to be characterized by increased P21 and decreased SOX9 expression (Fig. 2 F). Reactive oxygen species(ROS) is closely related to chondrocyte senescence. Accumulation of ROS resulted in mitochondrial dysfunction and significant chondrocyte senescence. Our results showed the fluorescence intensity of the experimental group was significantly higher than the control group, which indicated that ROS accumulation in FTH1-deficient chondrocytes (Fig. 2 G). These data demonstrate that loss of FTH1 promotes chondrocytes senescence and ECM degradation.

### Down regulation of FTH1 expression results in articular cartilage erosion, increasing sensitivity to ferroptosis

**Fig. 3:**
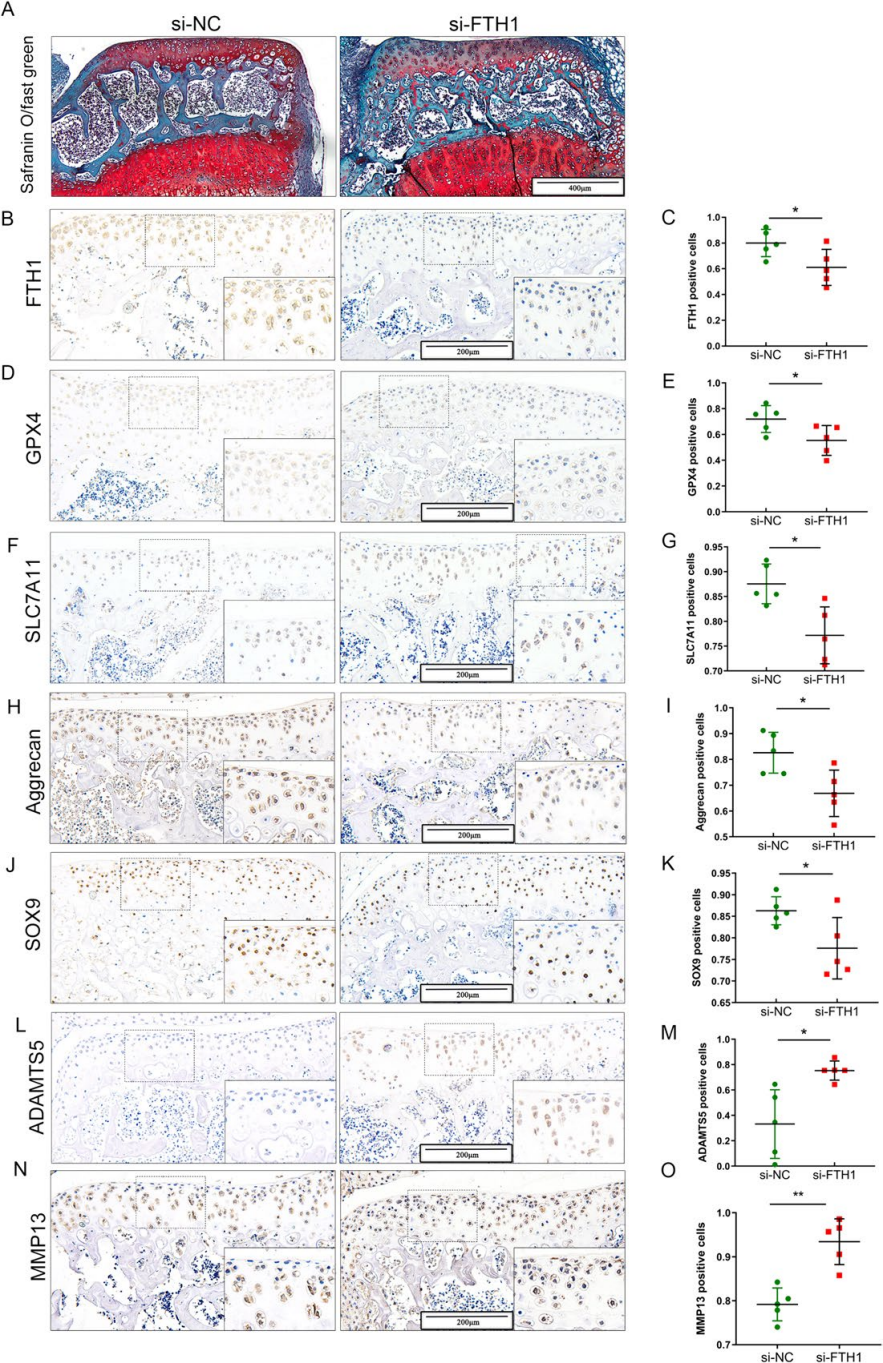
Down-regulation of FTH1 results in articular cartilage loss and the sensitive of ferroptosis increased. **A** Safranin O and Fast Green staining of cartilage explants from 3-week-old c57 mice which treated with si-NC and si-FTH1 for 72 h. Scale bar: 400 µm; Immunohistochemical staining and quantification of FTH1 (**B, C**) and GPX4 (**D, E**)and SLC7A11 (**F, G**) in cartilage explants from 3-week-old c57 mice which treated with si-NC and si-FTH1 for 72 h. *n* = 5 per group; Immunohistochemical staining and quantification of aggrecan(**H,I**) and SOX9(**J, K**) and ADAMTS5(**L,M**) and MMP13**(N,O)** in cartilage explants from 3-week-old c57 mice which treated with si-NC and si-FTH1 for 72 h. *n* = 5 per group *P < 0.05; Scale bar: 200 µm;

The effect of FTH1 deficiency on OA was evaluated in cartilage explants from 3-week-old male C57BL/6 mice treated with FTH1 siRNA for 72 h. Safranin O and Fast Green staining demonstrated fewer chondrocytes and more proteoglycan loss in cartilage explants treated with FTH1 siRNA for 72 h, compared to controls (Fig. 3 A). IHC demonstrated FTH1 to be decreased after treatment with FTH1 siRNA (Fig. 3 B, C). Moreover, GPX4 and SLC7A11expression significantly decreased with decreased FTH1 (Fig. 3 D-G). Previously, GPX4 and SLC7A11 were shown to be critical regulators of ferroptosis [10]. Ferroptosis and the expression of GPX4 are closely related to OA development, with knockdown of FTH1 decreasing GPX4 expression. Further, markers of chondrogenic matrix synthesis, SOX9 and aggrecan were slightly reduced while ADAMTS5 and MMP-13 increased obviously, as judged by IHC (Fig. 3 H–O). These results demonstrate FTH1 deficiency to result in cartilage loss and ECM degradation, inducing chondrocyte sensitivity to oxidative stress. Hence, down regulation of FTH1 promotes OA development by increasing the sensitivity of chondrocytes to ferroptosis, which disrupts ECM homeostasis.

### Over expression of FTH1 reduces cartilage loss and protects chondrocytes from DMM surgery

**Fig. 4:**
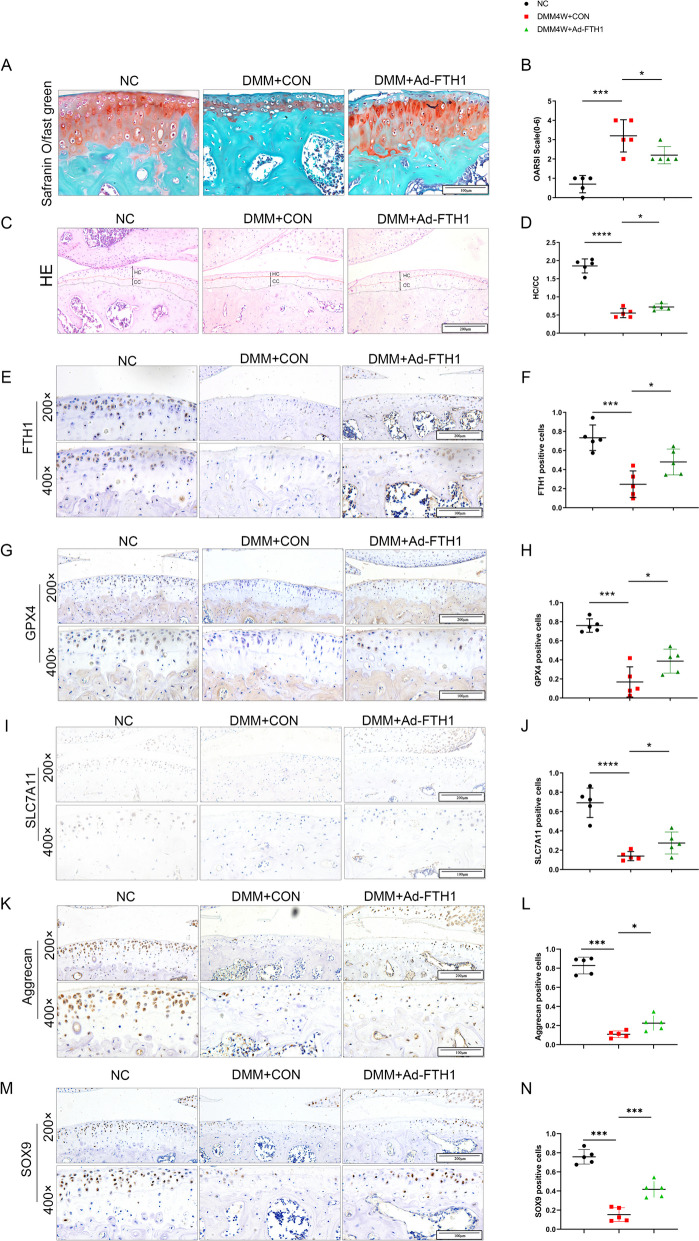
Overexpress FTH1 reduces cartilage loss and protect chondrocytes from DMM surgery. **A** Safranin O and Fast Green staining and quantification of sham-operated mice and mice treated with vehicle and Ad-FTH1 for 4 weeks after DMM surgery. *n* = 5 per group; Scale bar: 100 µm; **B** Osteoarthritis Research Society International (OARSI) grades of the joints described in A. *n* = 5 per group; **C-D** H&E staining of sham-operated mice and mice treated with vehicle and Ad-FTH1 for 4 weeks after DMM surgery; Scale bar: 200 µm; Immunohistochemical staining and quantification of FTH1 (**E, F**), GPX4 (**G,H**), SLC7A11 (**I, J**) aggrecan (**L, L**) and SOX9 (**M, N**); *n* = 5 per group; *P < 0.05, **P < 0.01, ***P < 0.001,****P < 0.0001; Scale bars: 200 µm (first row) and 100 µm (second row)

We have shown that FTH1 is associated with OA ECM degradation and ferroptosis. To assess a protective effect for FTH1, DMM male C57 mice that were 12 weeks of age were given intra-articular injections of adenovirus expressing FTH1. Mice that received the FTH1 adenovirus vector had considerably higher OA scores than negative controls. Over expression of FTH1 was characterized by a loss of proteoglycan, fewer chondrocytes, more hypertrophy chondrocytes, and increased cartilage erosion, which was lower than controls as judged by the OARSI scale and hematoxylin-eosin(HE) staining (Fig. 4 A-D). Previously, articular cartilage loss and erosion were demonstrated after knock down of FTH1. Significantly, after DMM surgery, FTH1, SLC7A11 and GPX4 levels were reduced in articular cartilage (Fig. 4 E- J). Markers of chondrogenic matrix synthesis, SOX9 and aggrecan, were also decreased after DMM surgery (Fig. 4 K, M). Further, decrements in FTH1,GPX4, SLC7A11, SOX9, and aggrecan were reversed by intra-articular injection of adenovirus expressing FTH1 (Fig. 4 E-N). These *in vivo* experiments demonstrated FTH1 to increase expression of anabolic markers and decrease chondrocyte sensitivity to ferroptosis after DMM surgery, suggesting decreased chondrocytes sensitivity and increased anabolism as the means by which to regulate cartilage homeostasis. As such, over expression of FTH1 reduces cartilage loss and maintains cartilage homeostasis by decreasing chondrocyte sensitivity to ferroptosis.

### FTH1 suppresses OA progression by inhibition of the MAPK pathway

**Fig. 5:**
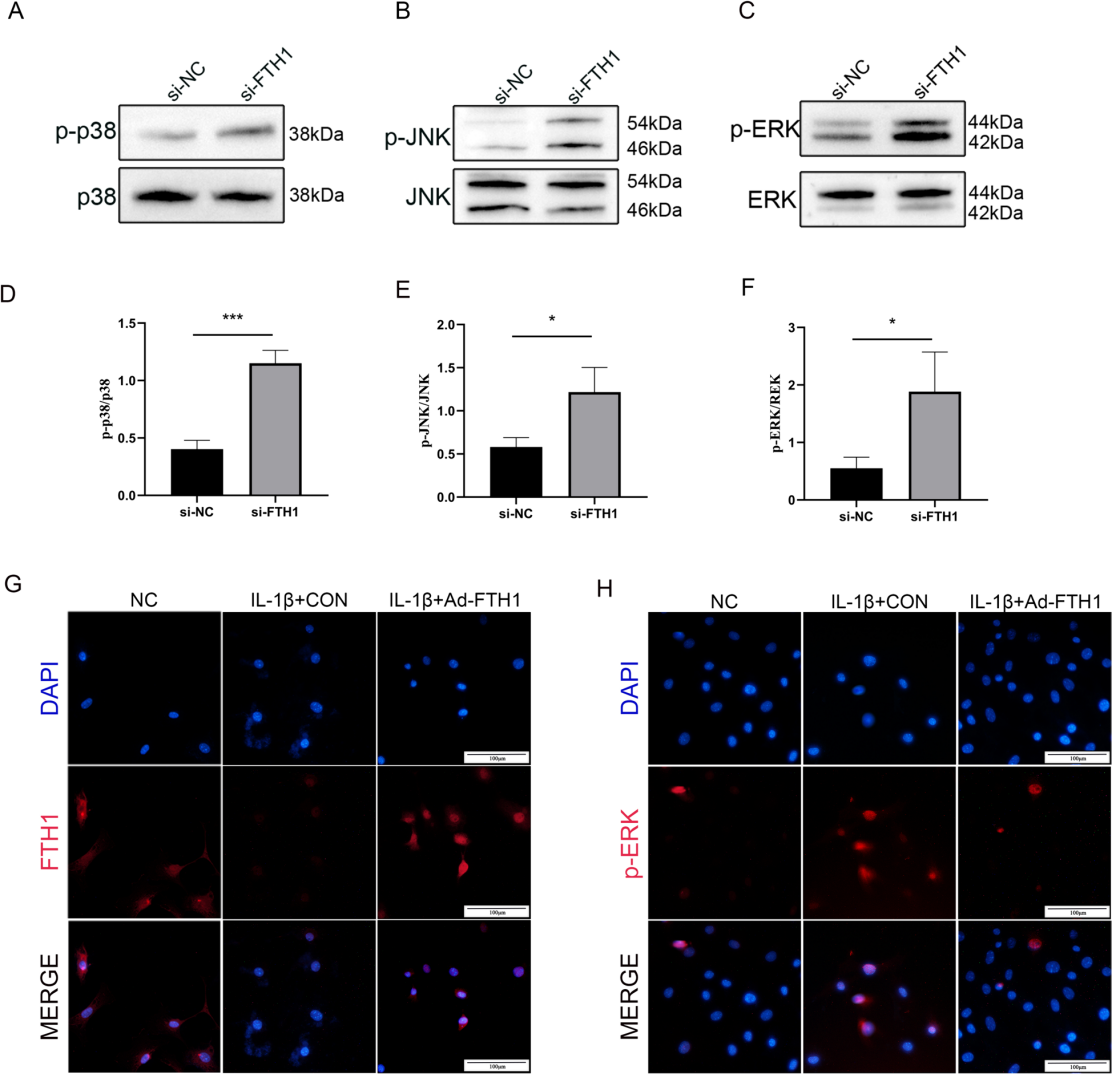
FTH1 suppress OA progression through inhibition of the MAPK pathway. Immunoblotting show that MAPK signal-related proteins p-P38/P38, p-JNK/JNK and p-ERK/ERK expression after knock down FTH1. **A-C** Gray scale analysis of p-P38/P38, p-JNK/JNK and p-ERK/ERK. *n* = 3 per group, *P < 0.05;**P < 0.01. **D-F G** Immunofluorescence images of FTH1 in primary chondrocytes, stimulated with IL-1β, vehicle and adenovirus- expressing FTH1. Scale bars: 100 µm. **H** Immunofluorescence images of phospho-ERK in primary chondrocytes, stimulated with IL-1β, vehicle and adenovirus- expressing FTH1. Scale bars: 100 µm

Although the association of FTH1 with OA has been established, the potential mechanism of FTH1 involvement in OA remains unclear. Proteomics analysis was performed on primary murine chondrocytes, both with and without FTH1 knockdown. Knockdown of FTH1 resulted in the upregulation of 1440 genes and the downregulation of 451 genes in chondrocytes. (Supplemental Fig. 2.A) KEGG pathway analysis revealed that FTH1 deficiency led to the promotion of N-Glycan biosynthesis, Parkinson's disease, and Huntington's disease, while inhibiting selenocompound metabolism and glycosaminoglycan biosynthesis. (Supplemental Fig. 2.B-D) Previous studies have demonstrated a correlation between changes in N-glycan expression and structure and the progression of osteoarthritis (OA). Additionally, N-glycan has been associated with the activation of gene expression of ERK1/2 [16, 17]. It’s widely accepted that the MAPK/ERK pathway plays an important role in many physiological processes including chondrocyte matrix degradation and macrophage M1 polarization [18]. In this study, we used western blotting to explore the effect of FTH1 deficiency on the MAPK pathway. We demonstrated the phosphorylation levels of P38, JNK and ERK, to be increased significantly after knockdown of FTH1 expression (Fig. 5 A-F). Treatment with adenovirus expressing FTH1 rescued decreased FTH1 expression (Fig. 5 G) and inhibited the phosphorylation of ERK (Fig. 5 H) in chondrocytes treated with IL-1β for 48 h. These results suggest that FTH1 deficiency induced chondrocyte senescence and ECM degradation, which resulted in articular cartilage erosion and OA exacerbation. As well, over expression of FTH1 reduced OA progression and inhibited phosphorylation of P38, JNK and ERK. Therefore, FTH1 suppress OA progression, at least in part by inhibition of the MAPK pathway.

## Discussion

OA is the most frequent type of arthritis and the major cause of persistent disability in individuals over 65 years of age [19, 20]. In this investigation, we provide evidence that FTH1 plays a critical role in modulation of chondrocyte senescence, ECM degradation, and ferroptosis during the development and progression of OA. We demonstrated that FTH1 deficiency activates the MAPK signaling pathway and thus exacerbates cartilage degeneration, chondrocyte senescence, ECM degradation, and ferroptosis during OA. Moreover, over expression of FTH1 reduced chondrocyte sensitivity to ferroptosis and increased markers of chondrocyte synthesis after DMM. Ferroptosis is a new form of cell death that was first discovered and confirmed in 2012. Understanding of its physiological mechanisms and potential as a therapeutic target are of continued scientific interest [7, 21]. Ferroptosis is a unique mode of cell death that has been extensively studied in cancer, inflammation, and degenerative diseases. Growing evidence suggests that ferroptosis drives the progression of degenerative diseases and inflammation. Some inhibitors of ferroptosis, such as antioxidants and iron chelators, lower cellular sensitivity to ferroptosis and reduce the release of inflammatory factors, further inhibiting ferroptosis [22].

Recent research has demonstrated iron chelators and antioxidants to reduce iron accumulation and cellular ferroptosis, thus inhibiting OA development [23, 24]. Lu et al [25] therapeutic targeting of ferroptosis may be a potential strategy for OA treatment. Ruiqing Lu, et al. found that a shortage of FTH1 induces ROS accumulation and sensitizes neuroblastoma N2A cells to ferroptosis [26]. Such evidence suggests that FTH1 deficiency induces iron overload and ROS accumulation, and sensitizing cells to ferroptosis [27, 28]. Of particular note, FTH1 homeostasis maintains intracellular labile Fe2 + , which suppresses ferroptosis [9]. Until now, the interaction of FTH1 with OA had not been reported. However, numerous issues remain and need to be resolved. There is no accepted gold standard for the measurement of ferroptosis. Further, knock down of GPX4 increases cellular sensitivity to ferroptosis [29]. Moreover, GPX4 is one of the three main inhibitors of ferroptosis and the expression of GPX4 is closely linked to ferroptosis [30]. Therefore, we chose to assess GPX4 and SLC7A11 expression as indexes of the presence or absence of ferroptosis in chondrocytes. We found that over expression of FTH1 reversed the GPX4 reduction observed after DMM surgery. These results suggest that over expression of FTH1 reduces chondrocyte sensitivity to ferroptosis and alleviates OA.

The cartilage microenvironment is important to the growth and maintenance of chondrocytes. Within the special environment of articular cartilage, the ECM provides oxygen and nutrients. With ECM homeostasis disruption, cartilage breaks down and erodes [31]. Therefore, we assessed the expression of collagen type II, SOX9, aggrecan, and ADAMTS5. Further, we determined the function of FTH1 in the development of OA. Lower levels of FTH1 were detected in OA cartilage, with knock down of FTH1 suppressing cartilage anabolism and accelerating chondrocyte senescence. Over expression of FTH1 slowed the progression of OA in DMM mice. Moreover, MAPK is a serine-threonine kinase family composed of p-JNK/JNK, p-ERK/ERK, and p-P38/P38, which regulate numerous cellular activities including: cell proliferation, differentiation, apoptosis, inflammation, and innate immunity. Activation of the MAPK signal pathway is highly related to inflammation and ECM degradation.

This study's potential limitations include: the use of an intra-articular adenovirus vector-coated FTH1 rather than transgenic mice, and an incomplete verification of the signaling pathway. Ferric oxide-hydroxide accounts for more than 20% of ferritin [32]. In this experiment, specific levels of ferric oxide-hydroxide in normal and osteoarthritic cartilage were not tested. However, a recent study has indicated that the levels of Fe2 + , Fe3 + , and total iron are higher in osteoarthritic cartilage compared to normal cartilage [15]. Additionally, it is worth noting that FTH1, a major iron storage protein, can lead to an increase in free iron when its expression is reduced or when ferritinophagy is intensified, thereby promoting ferroptosis [28, 33]. Overall, the role of FTH1 in restoration of joint function and delay of OA disease progression in OA is worth exploring in future studies. Interestingly, we noticed that both FTH1 and GPX4 were down regulated in the cartilage of OA patients and in the cartilage of mice after DMM. The expression of FTH paralleled that of GPX4 and it is widely accepted that GPX4 is one of the crucial markers of ferroptosis. So FTH1, as a potential marker of ferroptosis, requires and deserves subsequent in-depth study.

In summary, this study identified a role for FTH1 in OA and laid the foundation for future studies of the relationship between FTH1 and OA. Remarkably, the results indicate that intra-articular supplementation with FTH1 may be a means by which to delay the development of OA.

### Supplementary Information

**Supplementary Material 1.**

**Supplementary Material 2.**

## Data Availability

The dataset supporting the conclusions of this article is available at our institution contacting the corresponding author.

## Abbreviations

FTH1: Ferritin heavy chain 1
GPX4: Glutathione Peroxidase 4
DMM: Destabilization of the medial meniscus
SLC7A11: Solute Carrier Family 7 Member 11
ROS: Reactive oxygen species
ECM: Extracellular matrix
MMP13: Matrix Metallopeptidase 13
ADAMTS5: ADAM Metallopeptidase with Thrombospondin Type 1 Motif 5
SOX9: SRY-Box Transcription Factor 9
Col2a1: Collagen Type II Alpha 1 Chain
