# Supplementary material for: FTH1 protects against osteoarthritis by MAPK pathway inhibition of extracellular matrix degradation
Source: BMC Musculoskelet Disord. 2024 Apr 12;25:282. doi: 10.1186/s12891-024-07411-3 (PMC11010333; doi:10.1186/s12891-024-07411-3)
Supplement: Supplementary file 1 — Supplementary Material 1. [file 12891_2024_7411_MOESM1_ESM.doc]

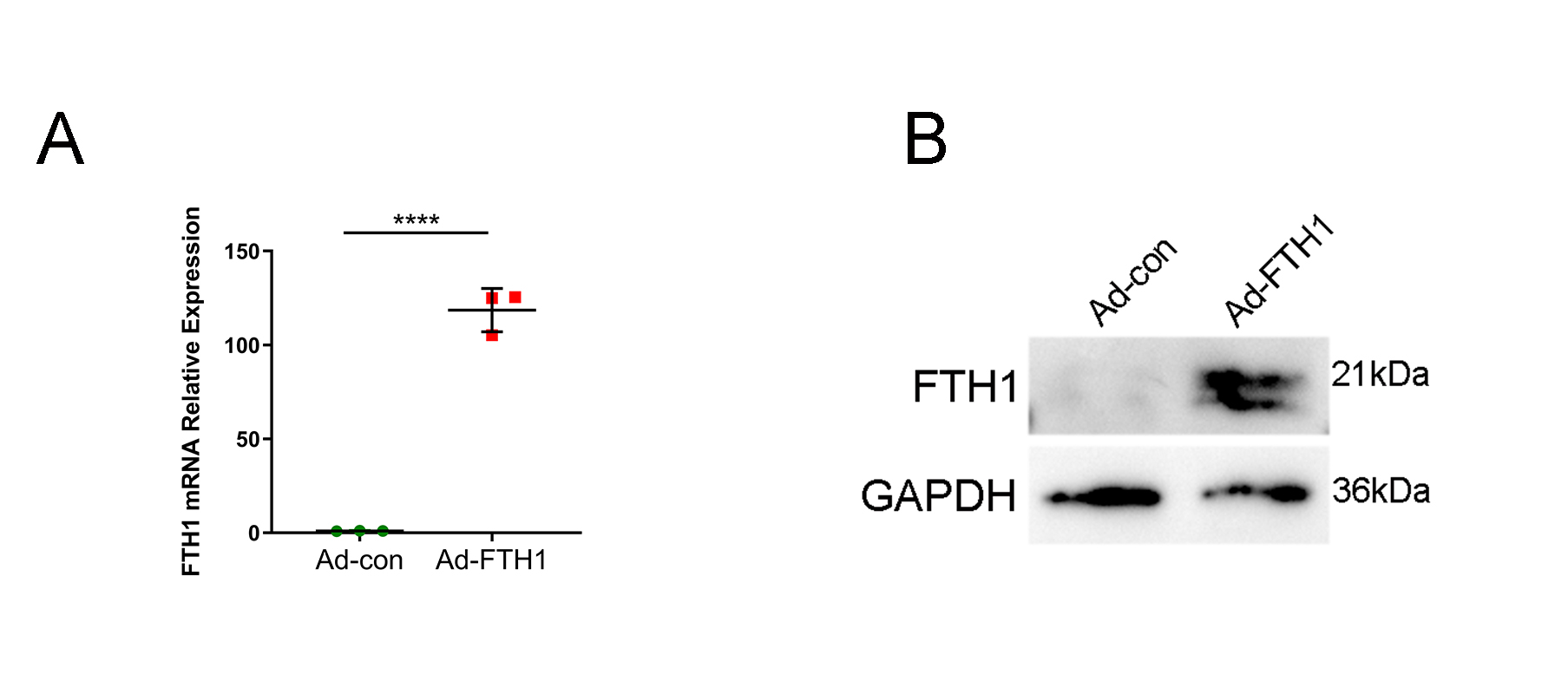


**Figure S1. FTH1 expression was significantly increased after transduction with Ad-FTH1**

**A** Relative mRNA expression level of FTH1 in primary murine chondrocytes which treated with Ad-FTH1 for 48 hours n=3, ****P < 0.0001 **B** Immunoblotting of FTH1 in primary murine chondrocytes which treated with Ad-FTH1 for 48 hours.


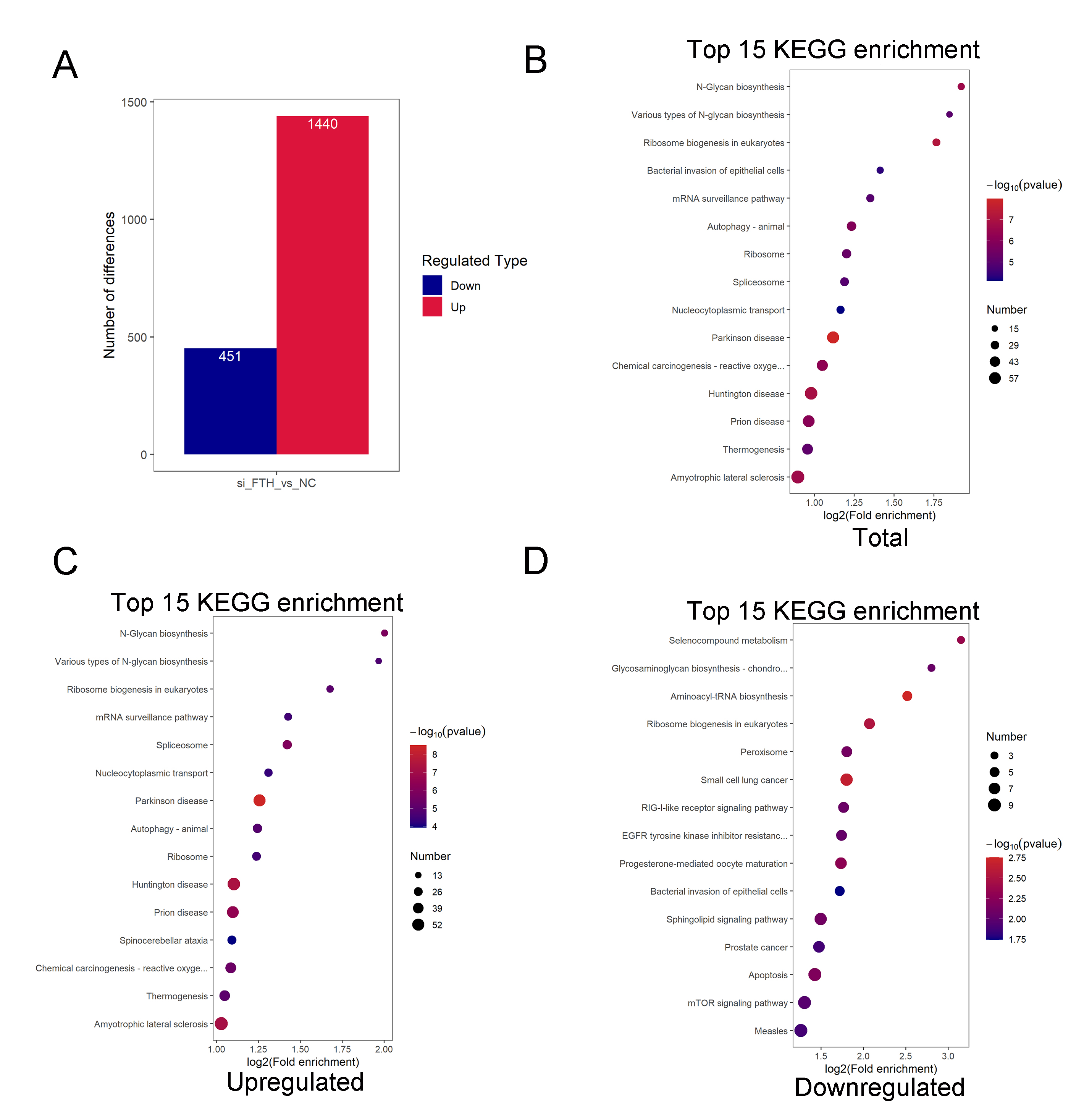


**Figure S2. KEGG pathway analysis of proteomics in chondrocytes with or without FTH1 knockdown. A** The number of differential genes. Kyoto Encyclopedia of Genes and Genomes (KEGG) analysis of total**(B)**, up-regulated**(C)**, and down-regulated**(D)**genes in si-FTH1 transcriptome. (Top 15)
