# Supplementary material for: FTH1 protects against osteoarthritis by MAPK pathway inhibition of extracellular matrix degradation
Source: BMC Musculoskelet Disord. 2024 Apr 12;25:282. doi: 10.1186/s12891-024-07411-3 (PMC11010333; doi:10.1186/s12891-024-07411-3)

Figure[1E] FTH1 Figure[1E] GAPDH


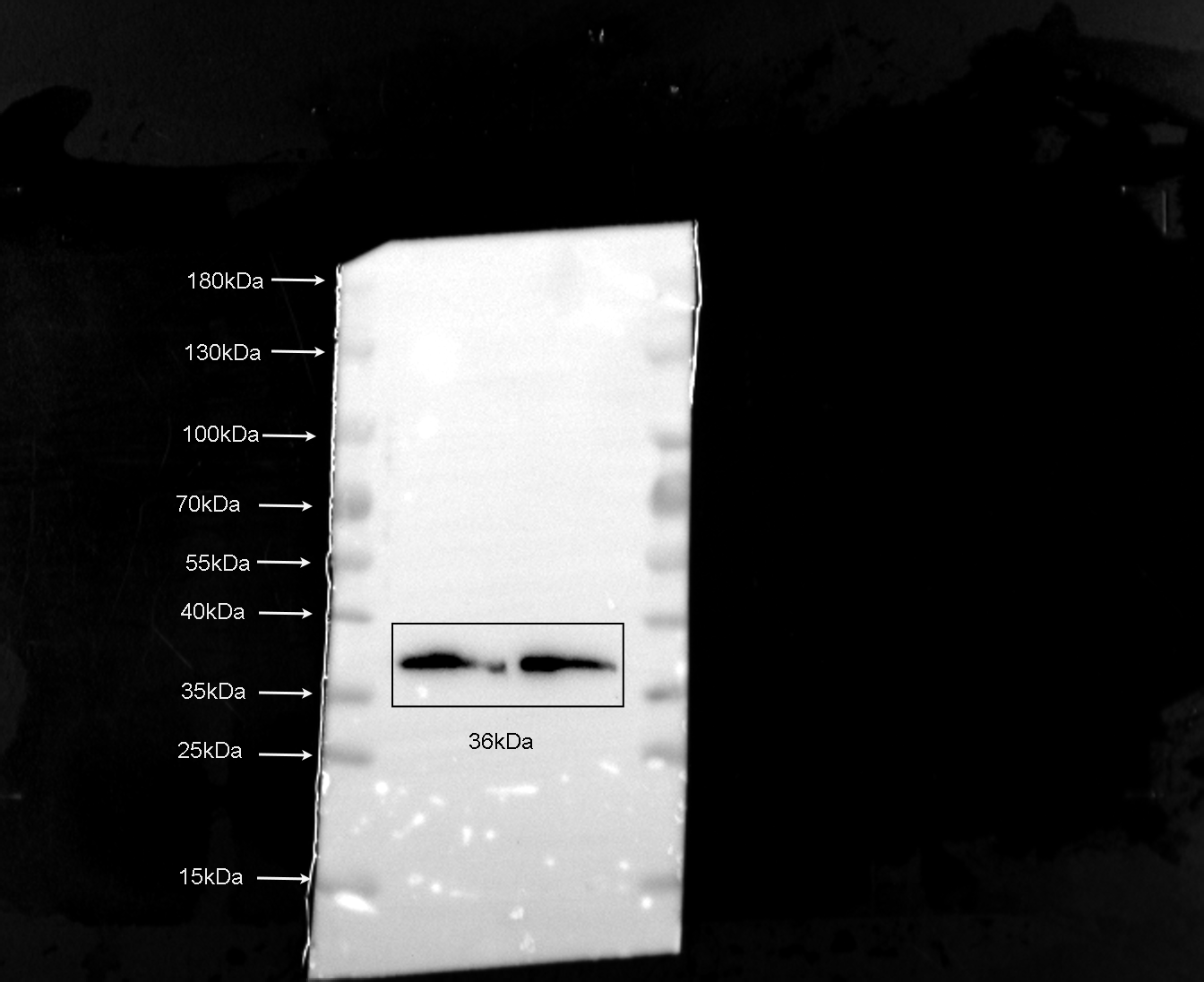

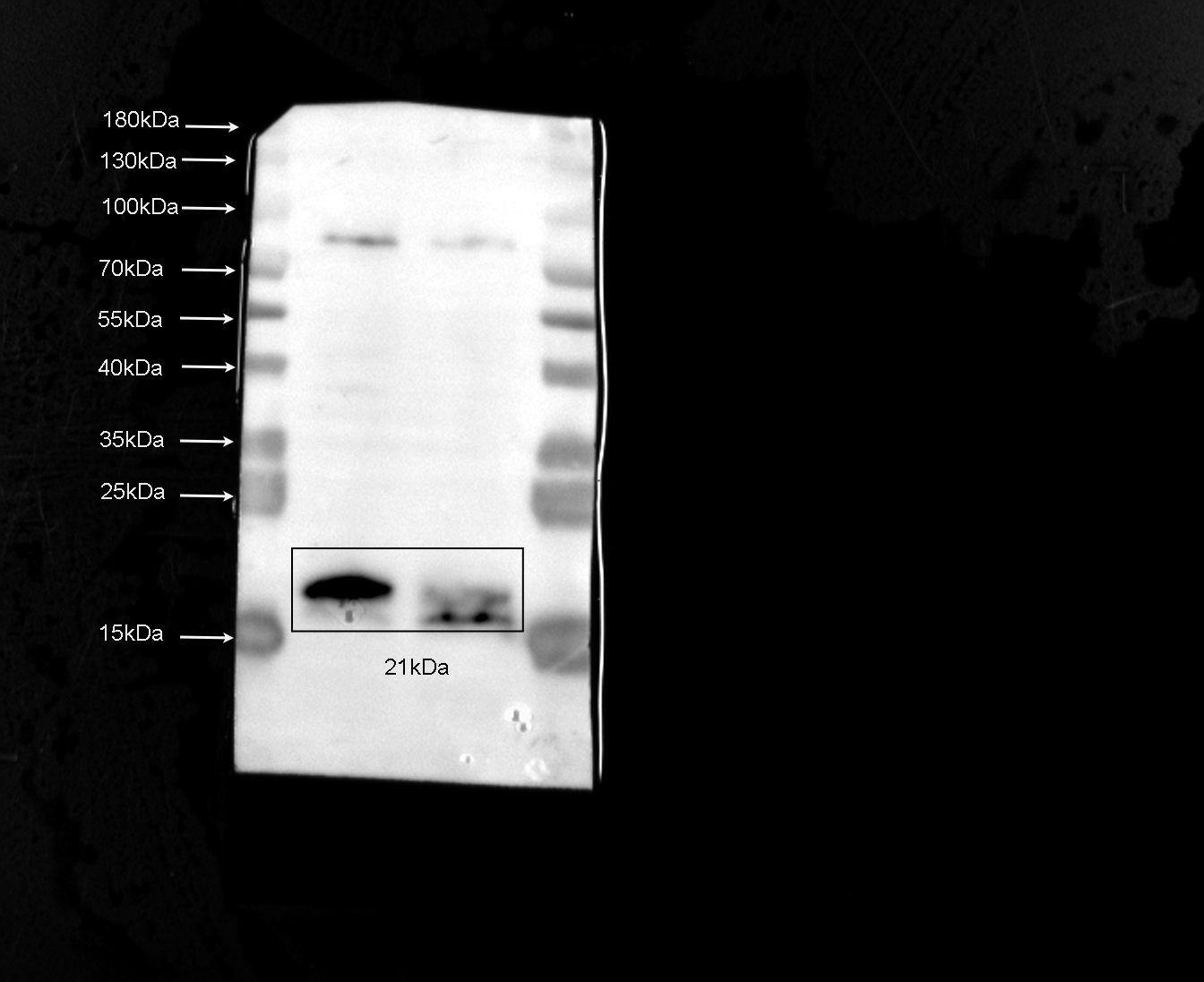


Figure[2F] GAPDH Figure[2F] FTH1


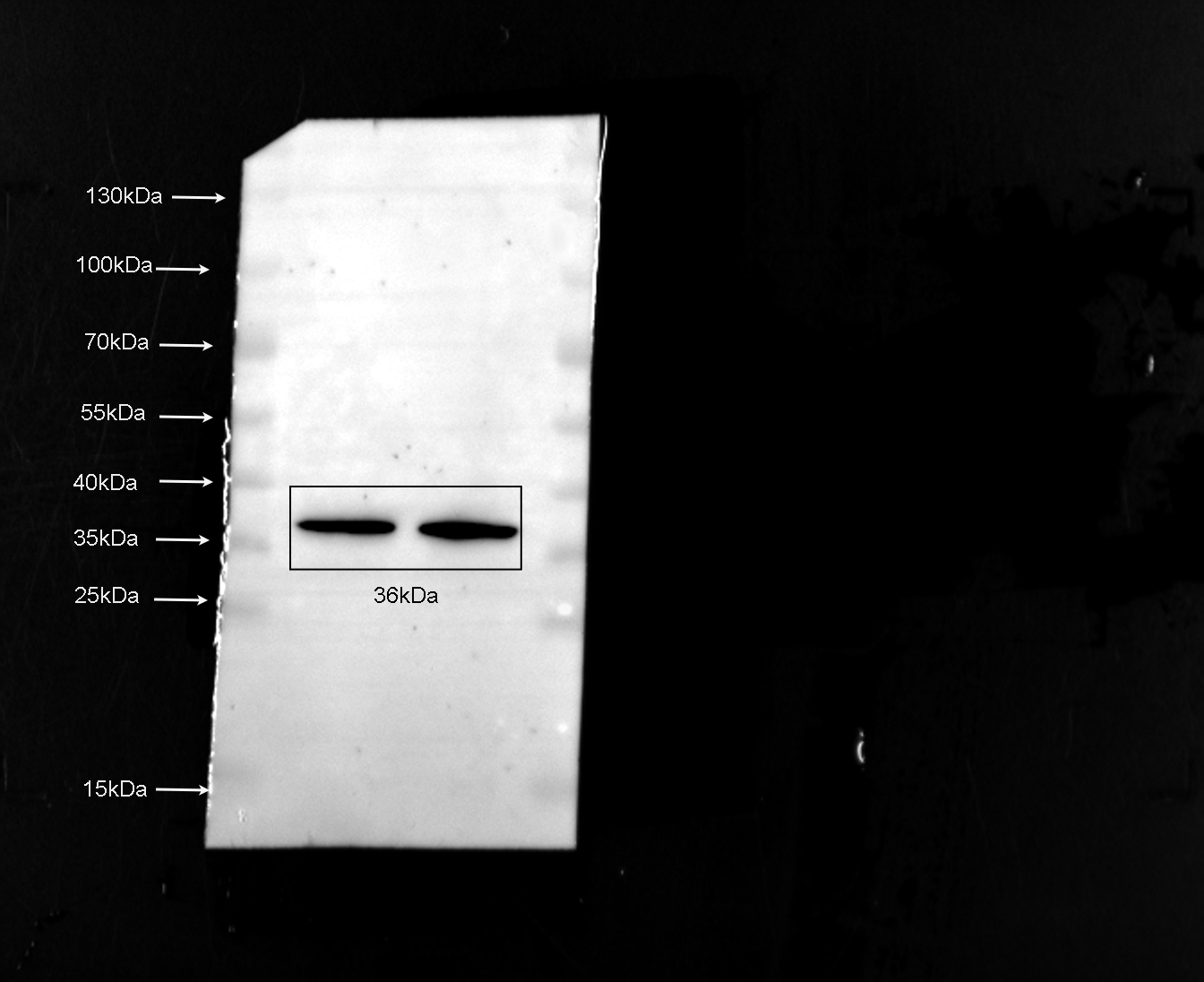

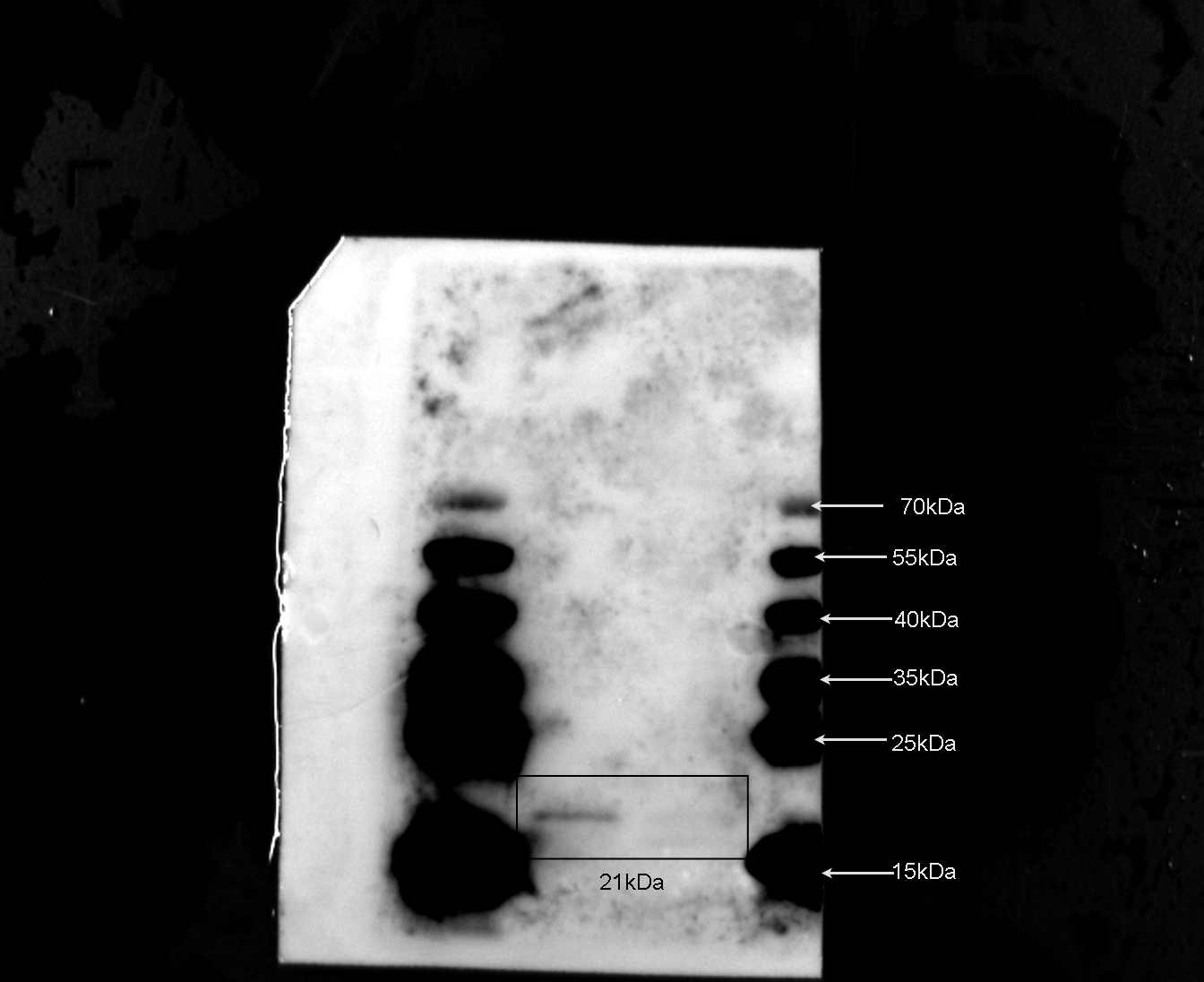


Figure[2F] P21 Figure[2F] SOX9


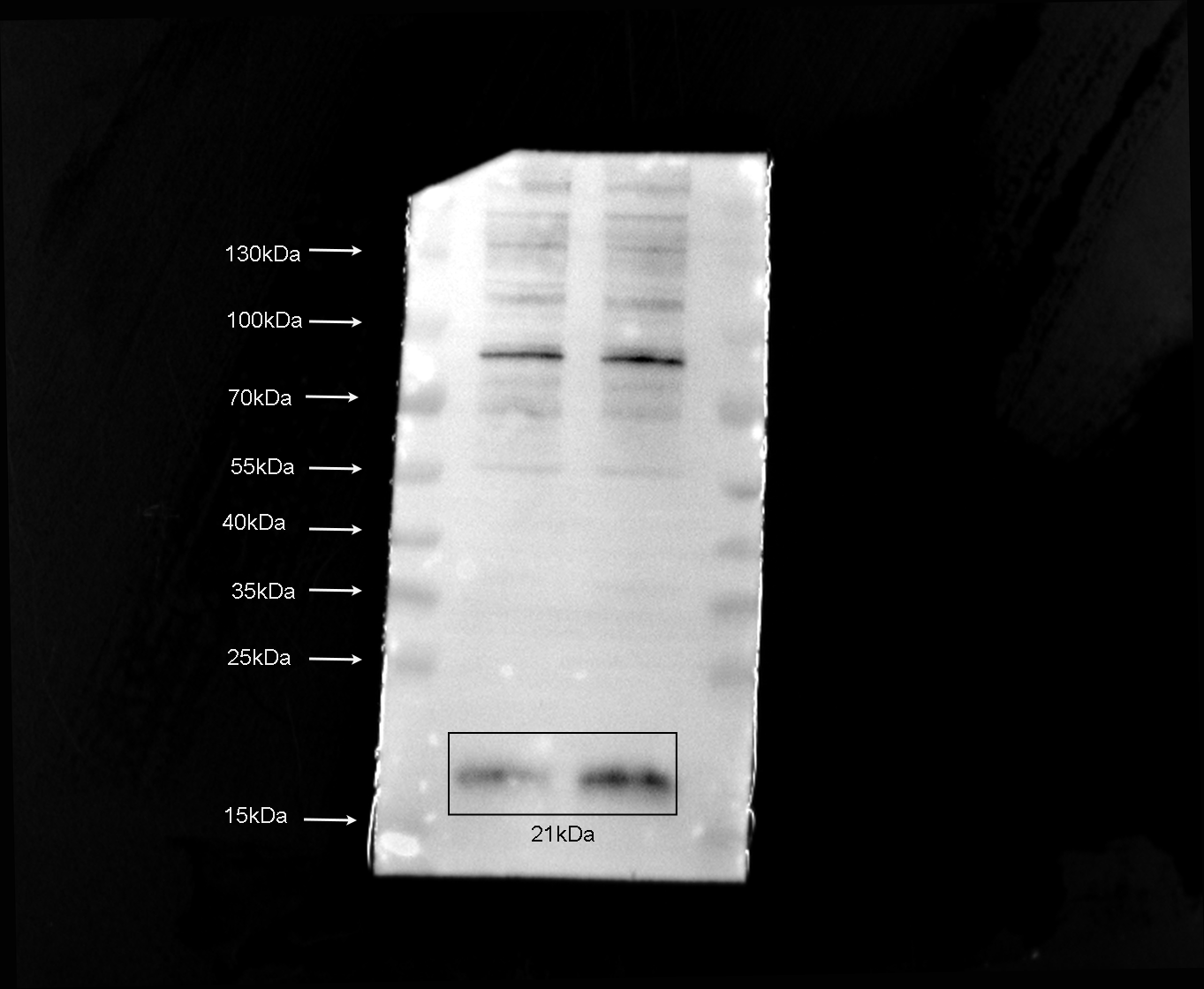

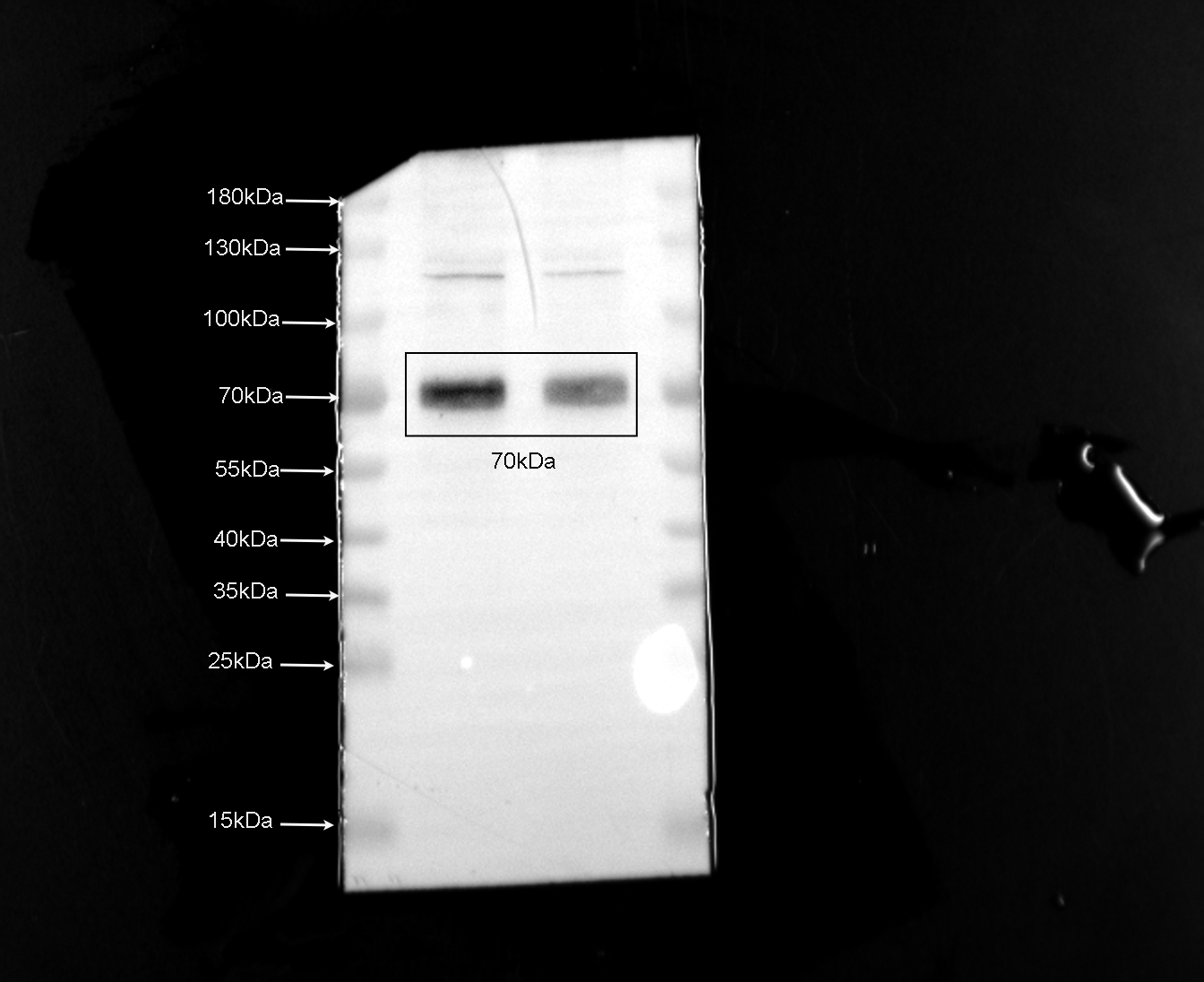


Figure[5A] JNK Figure[5A] p-JNK


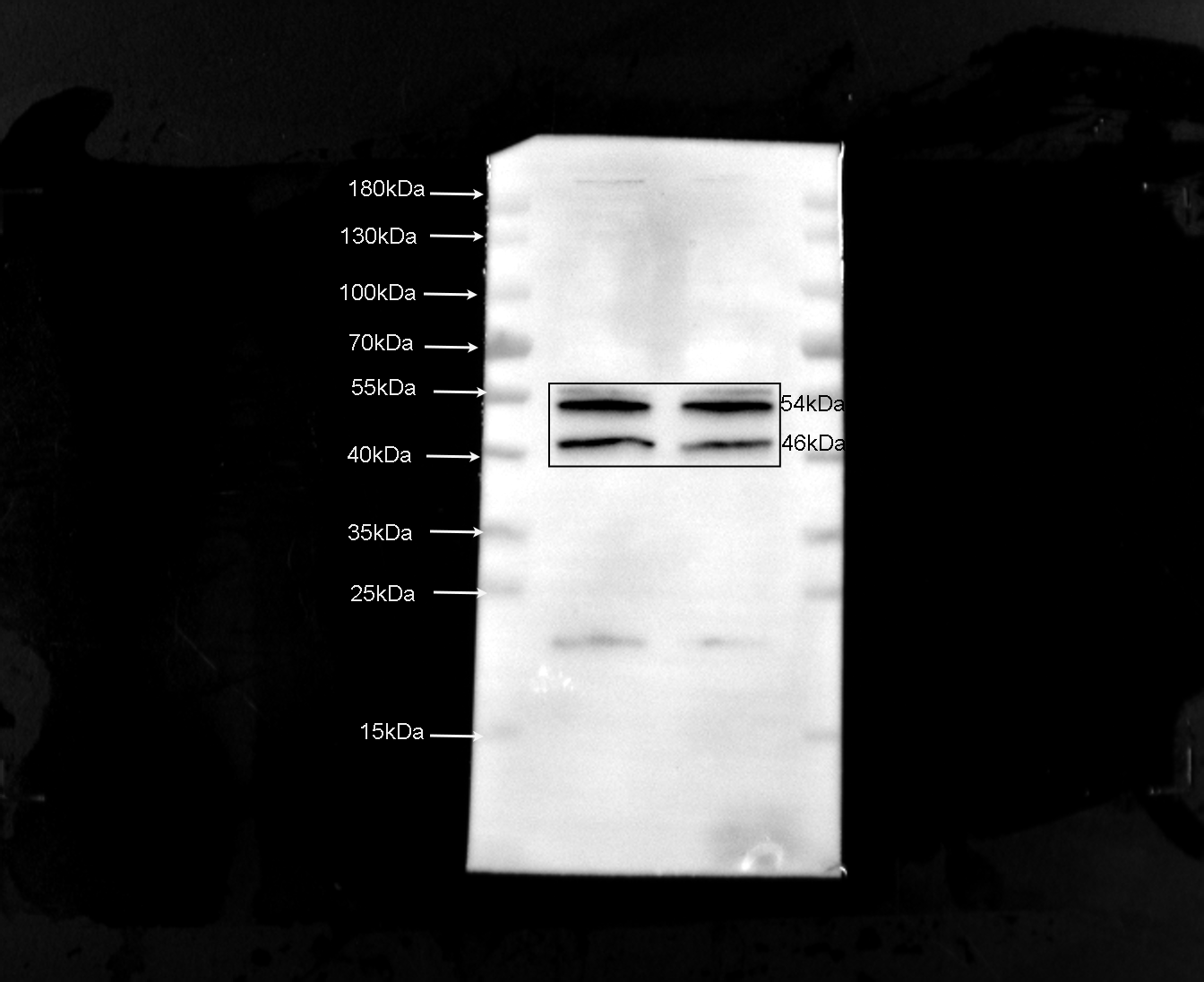

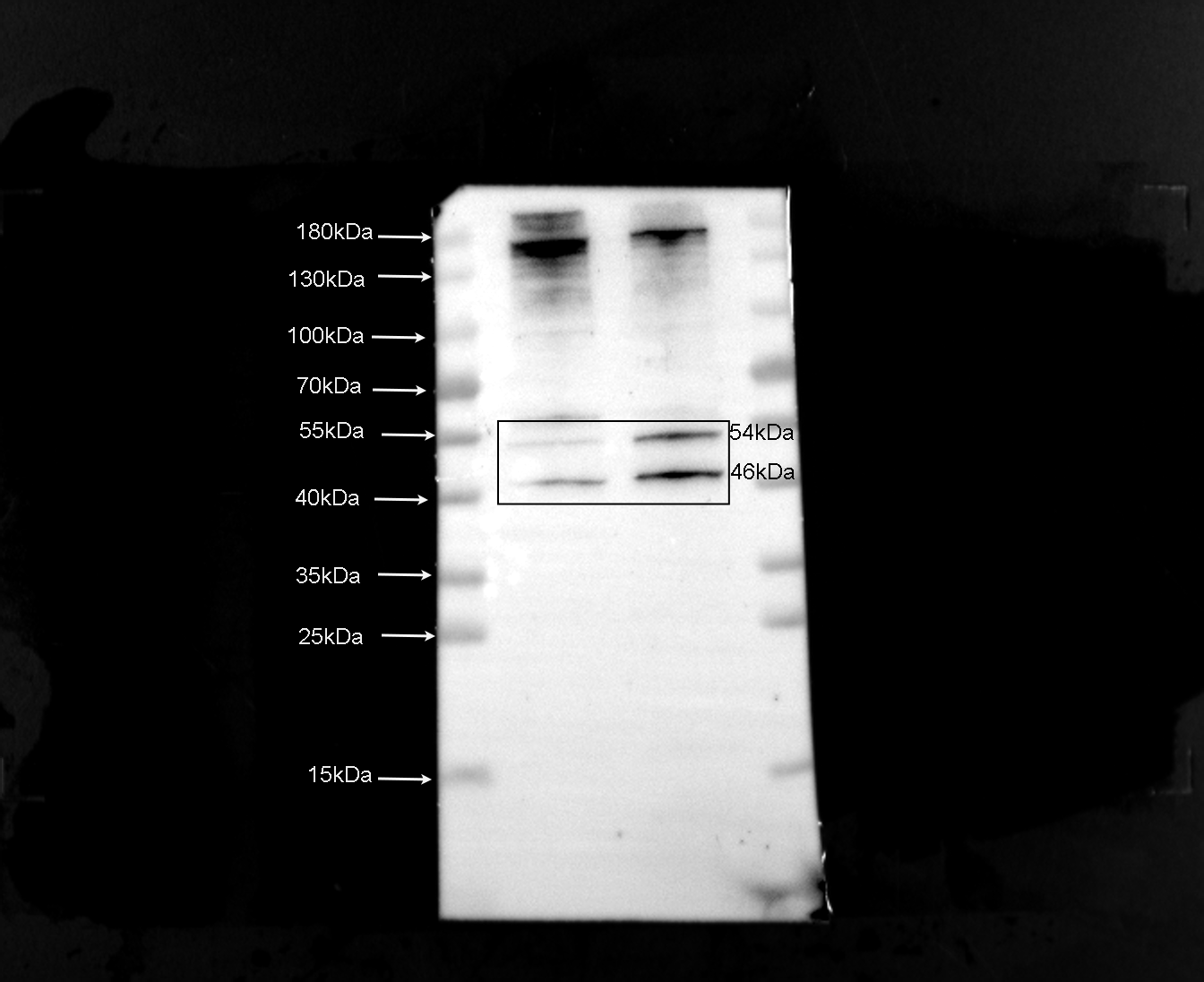


Figure[5B] P38 Figure[5B] p-P38


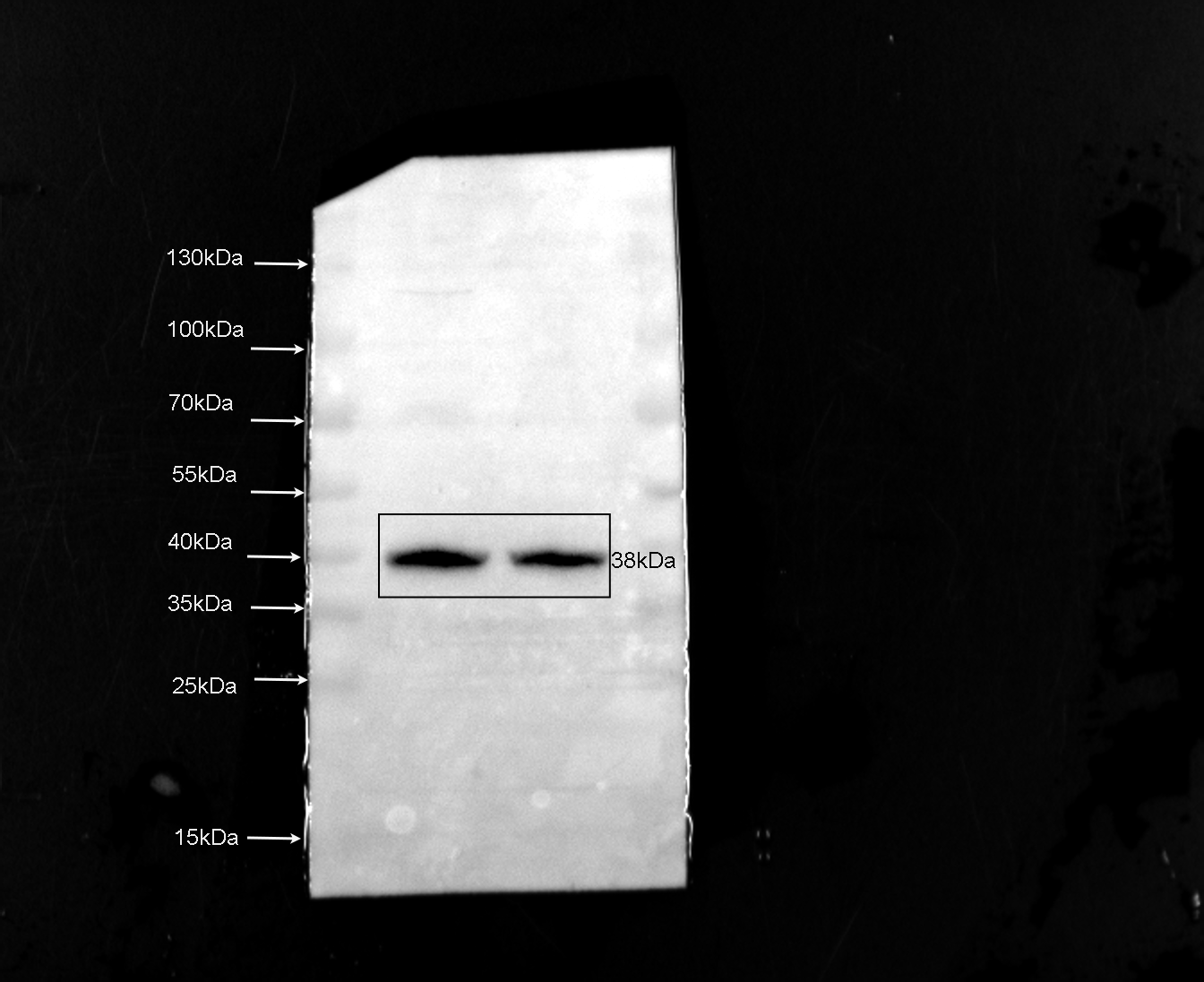

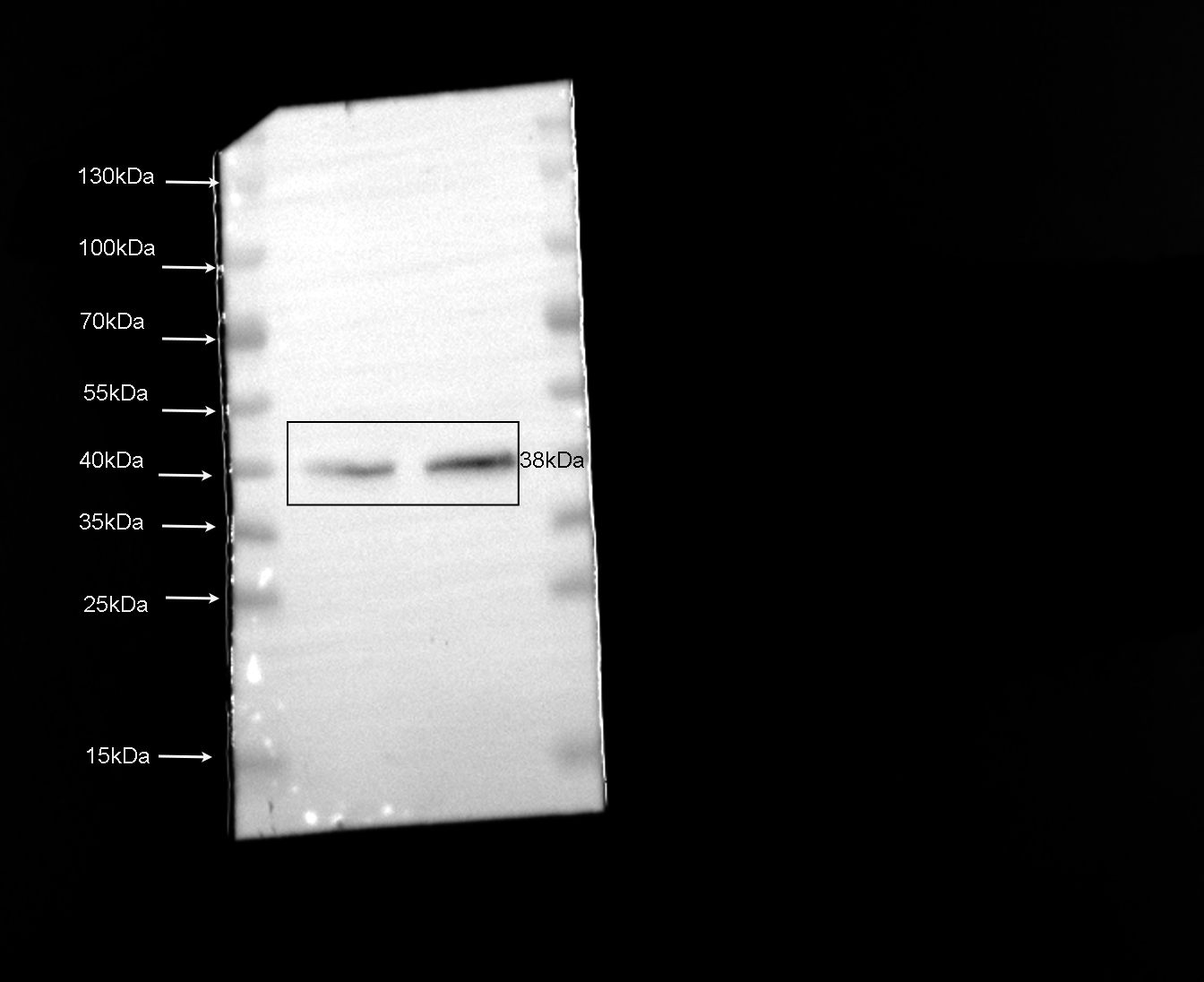


Figure[5C] ERK Figure[5C]p-ERK


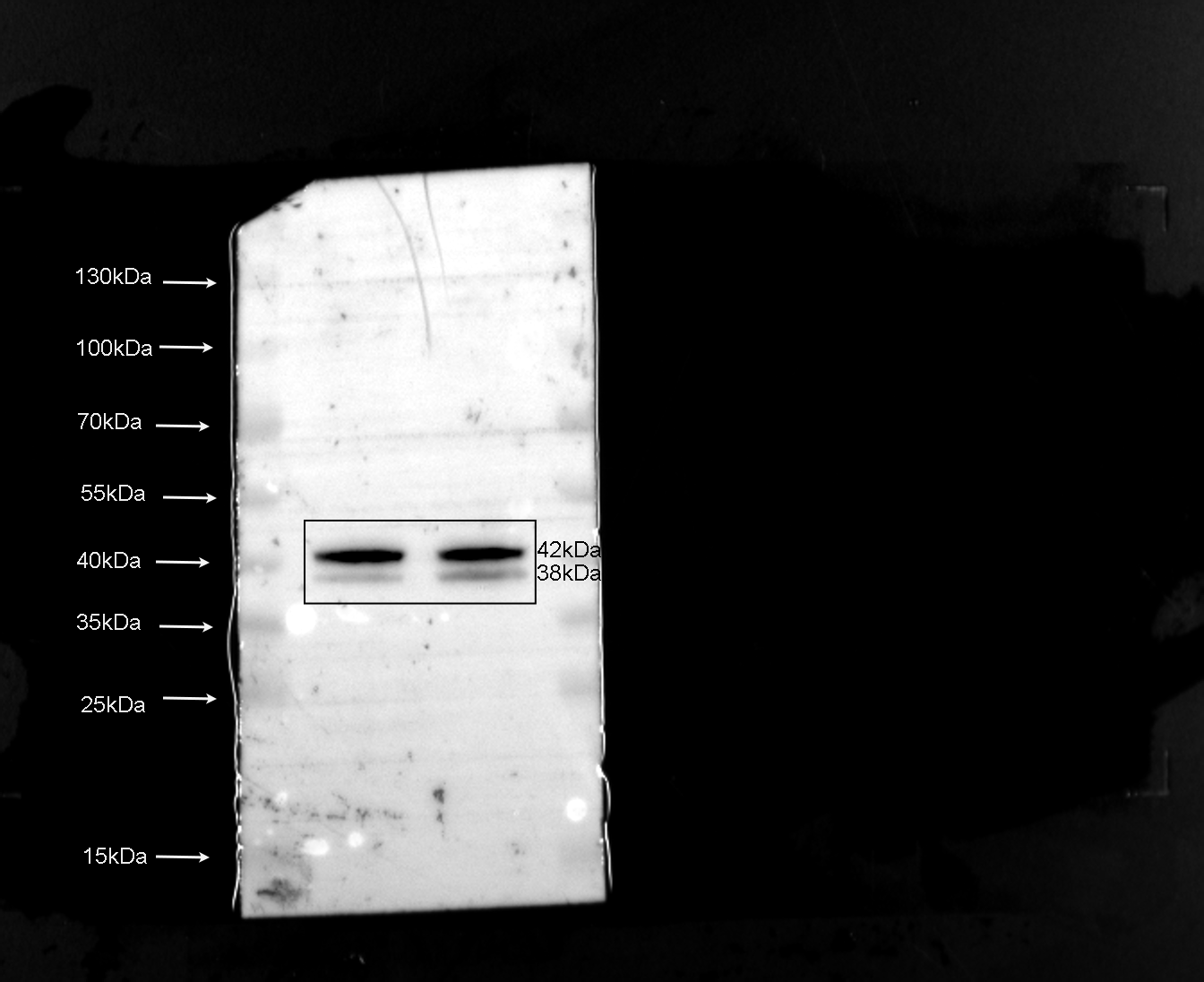

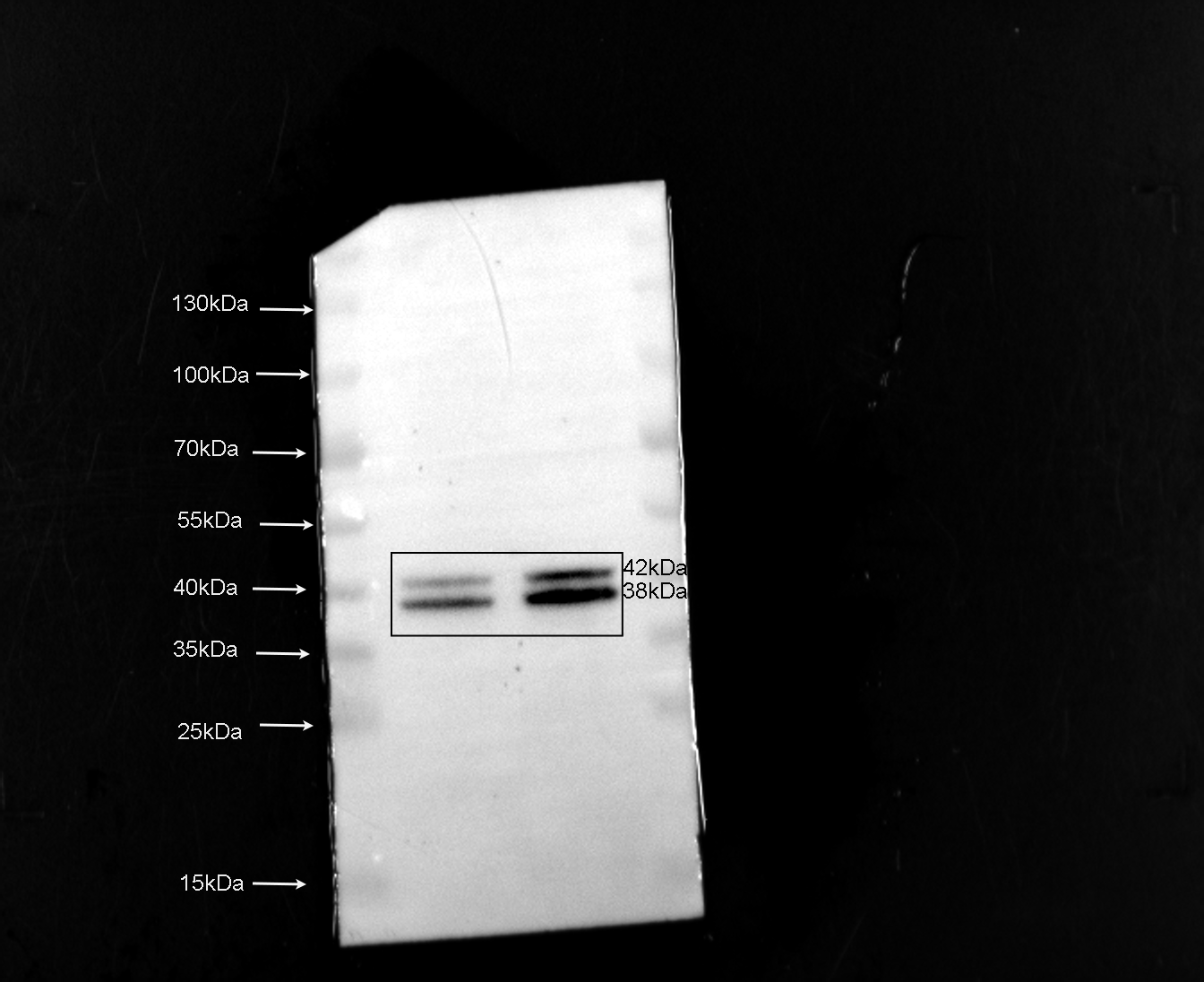


Supplemental Figure[1B] GAPDH Supplemental Figure[1B] FTH1


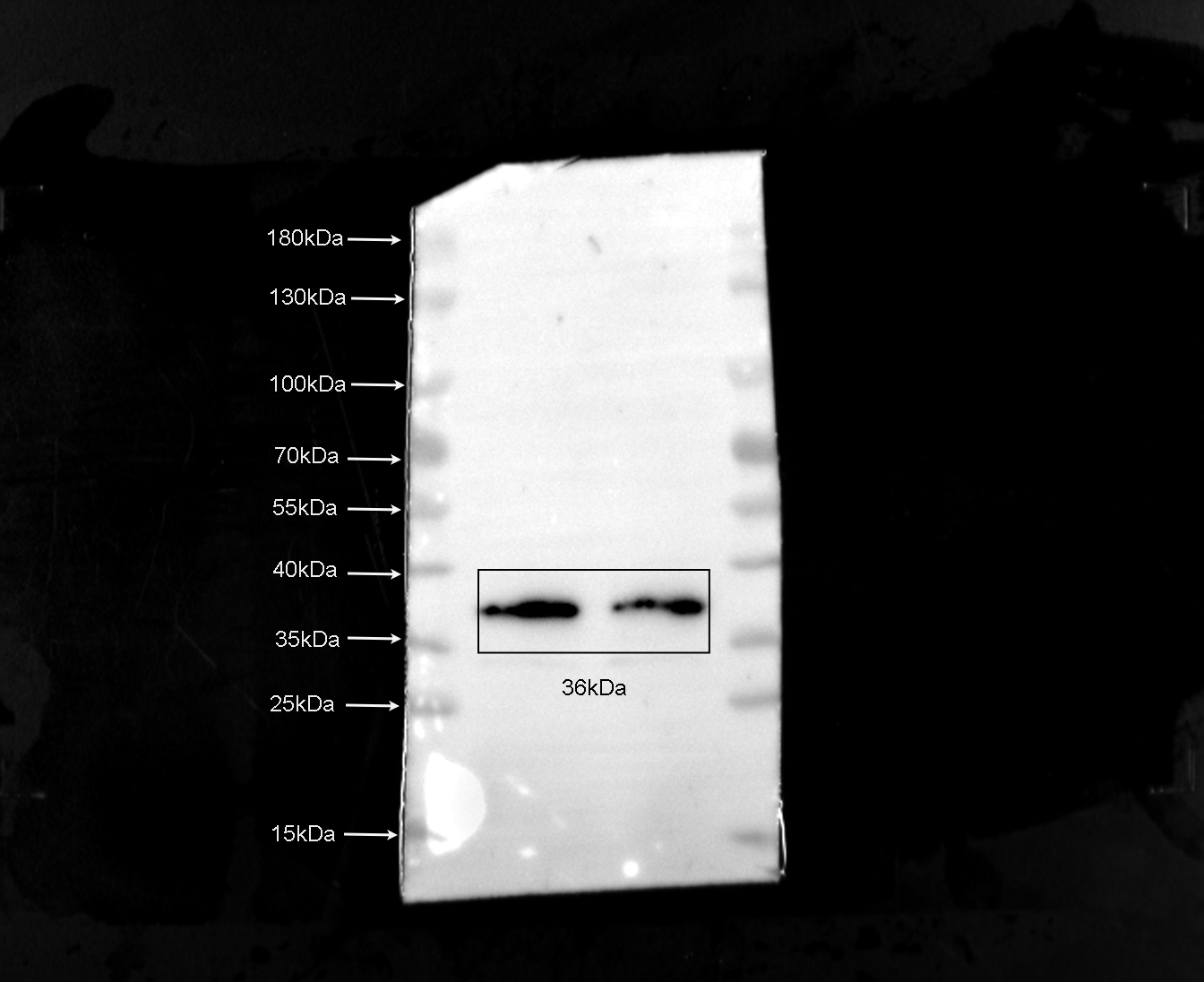

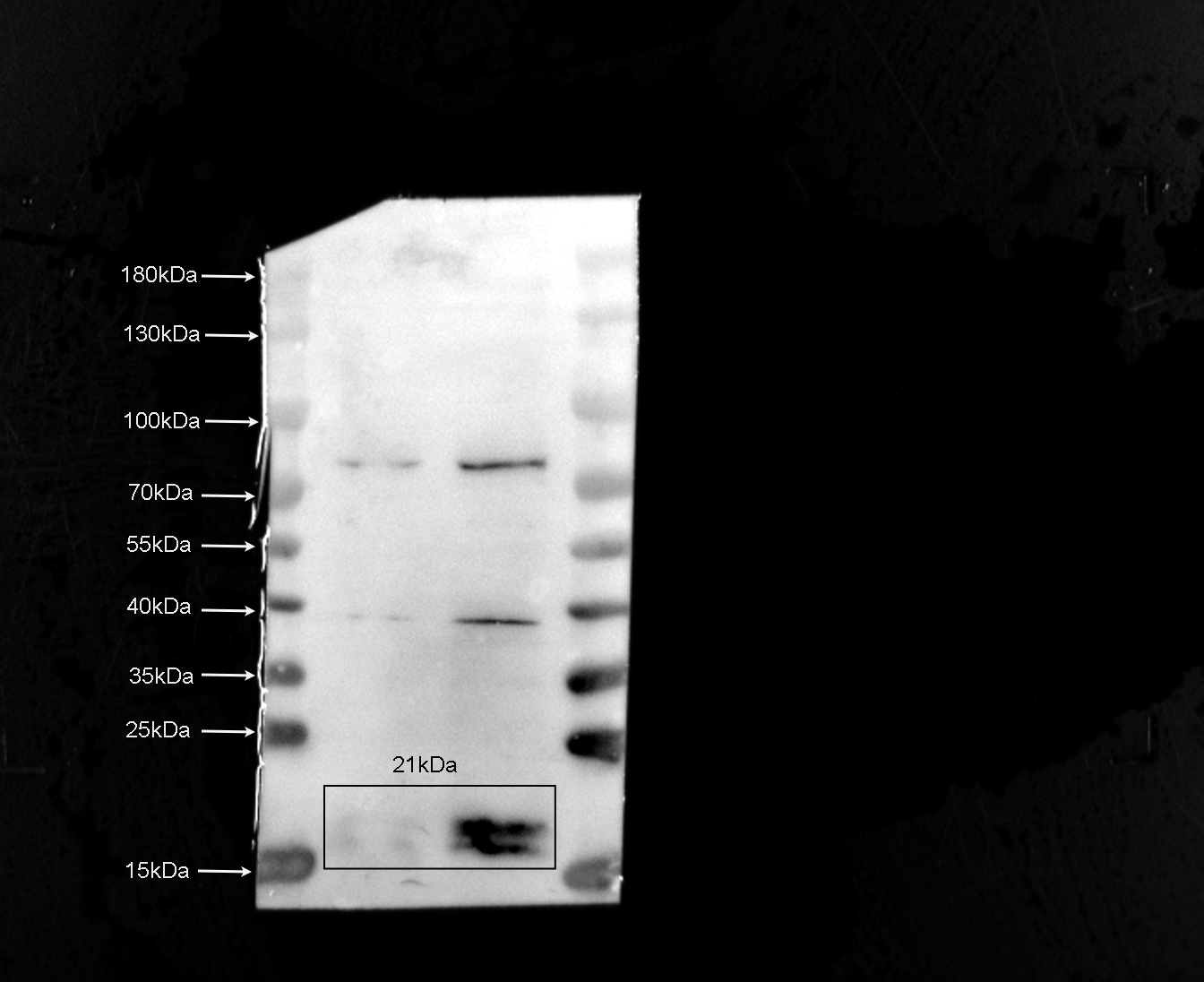

Supplement: Supplementary file 2 — Supplementary Material 2. [file 12891_2024_7411_MOESM2_ESM.doc]
